# Performance Comparison of Alternative Hollow-Fiber Modules for Hemodialysis by Means of a CFD-Based Model

**DOI:** 10.3390/membranes12020118

**Published:** 2022-01-20

**Authors:** Nunzio Cancilla, Luigi Gurreri, Gaspare Marotta, Michele Ciofalo, Andrea Cipollina, Alessandro Tamburini, Giorgio Micale

**Affiliations:** 1 Dipartimento di Ingegneria, Università di Palermo, Viale delle Scienze Ed. 6, 90128 Palermo, Italy; nunzio.cancilla@unipa.it (N.C.); michele.ciofalo@unipa.it (M.C.); andrea.cipollina@unipa.it (A.C.); alessandro.tamburini@unipa.it (A.T.); giorgiod.maria.micale@unipa.it (G.M.); 2 Medtronic^®^, Via Camurana 1, 41037 Mirandola, Italy; gaspare.marotta@medtronic.com

**Keywords:** hemodialysis, hollow-fiber membrane, solute clearance, computational fluid dynamics, porous media, Darcy permeability, ultrafiltration, mass transfer

## Abstract

Commercial hemodialyzers are hollow-fiber cylindrical modules with dimensions and inlet–outlet configurations dictated mostly by practice. However, alternative configurations are possible, and one may ask how they would behave in terms of performance. In principle, it would be possible to depart from the standard counter-flow design, while still keeping high clearance values, thanks to the increase in the shell-side Sherwood number (Sh) due to the cross-flow. To elucidate these aspects, a previously developed computational model was used in which blood and dialysate are treated as flowing through two interpenetrating porous media. Measured Darcy permeabilities and mass transfer coefficients derived from theoretical arguments and CFD simulations conducted at unit-cell scale were used. Blood and dialysate were alternately simulated via an iterative strategy, while appropriate source terms accounted for water and solute exchanges. Several module configurations sharing the same membrane area, but differing in overall geometry and inlet–outlet arrangement, were simulated, including a commercial unit. Although the shell-side Sherwood number increased in almost all the alternative configurations (from 14 to 25 in the best case), none of them outperformed in terms of clearance the commercial one, approaching the latter (257 vs. 255 mL/min) only in the best case. These findings confirmed the effectiveness of the established commercial module design for the currently available membrane properties.

## 1. Introduction

Hemodialysis is a membrane-based process in which solute removal occurs mainly via diffusion through the membrane [1]. Two fluids are involved in the hemodialysis process: the blood, rich in undesired solutes, which is purified by a rinsing solution called dialysate, from which it is separated by the membrane. The dialysate is a dilute solution of electrolytes and, sometimes, glucose. This solution contains sodium, magnesium and chloride ions at the same concentration as in normal plasma. To buffer the pH of the solution, bicarbonate or acetate is also added. Sometimes a fine-tuning of the dialysate composition is performed to calibrate the treatment on the patient [2,3]. The core of the process is represented by the semi-permeable membrane, which allows the removal of toxic substances and metabolic wastes, such as urea or creatinine, but prohibits the passage of proteins and cells from the blood to the dialysate [4].

**Figure 1:**
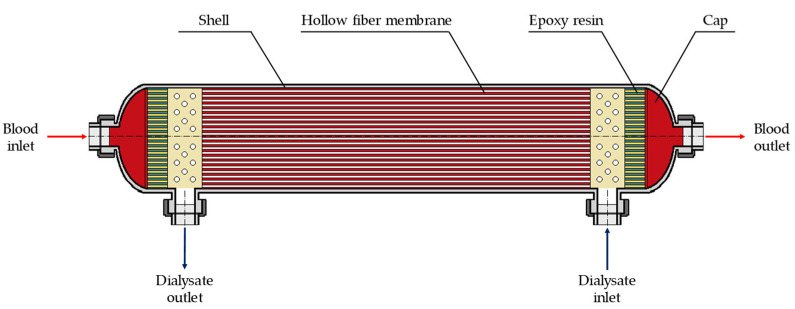
Schematic representation of a commercial hollow-fiber module for hemodialysis.

The devices commonly used in hemodialysis therapies are cylindrical modules filled with hollow polymeric fibers, called hemodialyzers. The typical sizes of a module range from 2 to 5 cm in diameter and from 15 to 25 cm in length. The housing usually consists of a transparent polymeric material (e.g., polycarbonate or polypropylene) enclosing a bundle of several thousand (~8000–16,000) hollow fibers. The module is also provided with inlet and outlet openings for the two fluids (Figure 1) [5].

On the lumen-side, the blood flows inside the hollow fibers while, on the shell-side, the dialysate flows outside the bundle in counter-current mode. The main driving force for mass transfer is the difference in solute concentration between the two compartments. The solutes are removed from blood to dialysate through the wall of the membrane by means of a diffusive-convective mass transfer process, since also the pressures of blood and dialysate are different [6].

The modelling of fluid flow and/or mass transfer in hollow-fiber hemodialyzers has involved many research groups being devoted to this purpose in the last 40 years. In fact, a really accurate model may be a useful tool for predicting the best operating conditions and the optimal design of a hemodialyzer, thus saving time and also reducing experimental costs.

Many studies on the modelling of the hemodialysis process have been conducted, focusing their attention on various aspects. Many researchers studied the simultaneous effect of diffusion and convection on mass transfer [7,8,9,10,11,12]. In particular, Chang and Lee [13], using a one-dimensional (1-D) model, proposed a simple equation for predicting the enhancement in clearance allowed by ultrafiltration in addition to simple hemodialysis. Also Jaffrin et al. [14] studied the interaction between diffusive and convective mass transfer in hemodialysis by employing a 1-D model: they demonstrated that the overall clearance is less than the sum of clearances from pure dialysis and ultrafiltration taking place separately. Another 1-D model developed by Legallais et al. [15] takes into account, besides ultrafiltration, phenomena such as concentration polarization and oncotic pressure.

Some researchers used tomographic techniques, e.g., single-photon emission computed tomography (SPECT) [16] or magnetic resonance imaging (MRI) [17], for validating their two-dimensional (2-D) models. Other 2-D models by various authors aimed at studying the effects on mass transfer of the protein adsorption on the inner surface of the membrane [18], the impact of various parameters on solute removal [19] and the influence of the shell-side flow on the performance [20,21].

In the last years, three-dimensional (3-D) models have been developed, based on the concept that the fiber bundle can be treated as two interpenetrating porous media. For example, Ding et al. [22] used their model to investigate the effects on mass transfer of inlet and outlet geometrical structures and of the distribution tabs [23]. Also Cancilla et al. [24] developed a model that falls into this category for studying the influence of the most relevant parameters and of the operating conditions on the dialyzer’s efficiency.

Despite the large number of scientific papers regarding various aspects of hemodialysis modelling, to the best of the authors’ knowledge, a comparative study on the influence on the performance of alternative geometries compared to the commercial ones is still lacking. The purpose of this work is to apply the model of two interpenetrating porous media that was previously developed to predict how the geometrical configuration of the dialyzer can affect not only overall parameters, such as solute clearance and ultrafiltration contribution, but also dialysate flow distributions and pressure drops in the dialyzers.

## 2. Materials and Methods

### 2.1. Computational Model

The model [24], developed by means of the finite volume code Ansys CFX^®^, simulates hemodialysis on hollow-fiber membrane modules by using the concept of two interpenetrating porous media [22,23]. Blood and dialysate are assumed to flow through two equivalent porous media, each characterized by a proper porosity and by two values of the Darcy permeability (axial and transversal). These latter values have been experimentally determined for commercial dialyzers. The characterization of the porous media can be managed in the code via the addition of the following momentum source terms to the right-hand side of the momentum equations:

(1) $$S_{M,\;x}=-\frac{\mu }{K_{t}}\;u_{x}$$

(2) $$S_{M,y}=-\frac{\mu }{K_{t}}\;u_{y}$$

(3) $$S_{M,z}=-\frac{\mu }{K_{z}}\;u_{z}$$

where *<u_x_>*, *<u_y_>* and *<u_z_>* are the superficial velocities along the *x*, *y* and *z* directions, respectively, *K_z_* and *K_t_* the axial and transversal Darcy permeabilities and *μ* is the viscosity.

Ultrafiltration is accounted for by adding to the right-hand side of the continuity equation the following mass source term:

(4) $$S_{M}=\pm \frac{A_{ext}}{V_{tot}}\cdot \rho \cdot L_{p}\cdot \left(p_{B}-p_{D}-p_{onc}\right)$$

in which the “minus” sign holds for blood and the “plus” sign for dialysate. In Equation (4), *ρ* is the density, *A_ext_* is the external surface area of the hollow fibers, *V_tot_* is the module’s volume, *L_p_* is the hydraulic permeability of the membrane, *p_B_* and *p_D_* are the lumen-side and dialysate-side pressures and *p_onc_* is the oncotic pressure of proteins in the blood.

Likewise, in order to account for ultrafiltration solute mass flux, the solute’s scalar transport equation is rewritten by adding a source term to the right-hand side:

(5) $$S_{C}=\pm \frac{A_{ext}}{V_{tot}}\cdot j$$

with, again, the “minus” sign applying to blood and the “plus” sign to dialysate. The term *j* is the solute mass flux per unit membrane area (in mol m^−2^ s^−1^) so that *S_C_* represents the solute mass flux per unit volume (in mol m^−3^ s^−1^).

In its turn, the flux *j* is expressed as:

(6) $$j=L_{p}\cdot \left(p_{B}-p_{D}-p_{onc}\right)\cdot \left(1-\sigma \right)\cdot C_{s,M}+U\cdot \left(C_{B}-C_{D}\right)$$

where *σ* is Staverman’s reflection coefficient and *C_s,M_* is the solute concentration in the fluid crossing the membrane, computed as the arithmetic mean of the lumen-side and dialysate-side bulk concentrations, *C_B_* and *C_D_*. *U* is an overall mass transfer coefficient, which is expressed as follows:

(7) $$U=\frac{1}{\frac{1}{k_{B}}+\frac{1}{k_{M}}+\frac{1}{k_{D}}}$$

In which *k_B_*, *k_M_* and *k_D_* are the mass transport coefficients of blood, membrane and dialysate, respectively. The blood- and dialysate-side mass transfer coefficients, *k_B_* and *k_D_*, are calculated as:

(8a) $$k_{B}={\mathrm{Sh}}_{B}\frac{D_{B}}{d_{i}}$$

(8b) $$k_{D}={\mathrm{Sh}}_{D}\frac{D_{D}}{d_{h}}$$

In Equation (8), *D_B_* and *D_D_* are the solute diffusivities in blood and dialysate, respectively, *d_i_* and *d_h_* are the internal diameter of the hollow fibers and the hydraulic diameter of the fiber bundle, respectively, and Sh*_B_* and Sh*_D_* are the lumen-side (blood) and shell-side (dialysate) Sherwood numbers, respectively.

For Sh*_B_* a value of 4, intermediate between the values for uniform wall concentration and uniform wall mass flux for parallel flow in cylindrical ducts, is used in the simulations.

For Sh*_D_* the following Equation (9) is used:

(9) ShD=9.85 (1+1.41·Ret0.38)

which expresses Sh*_D_* as a function of the shell-side cross-flow Reynolds number Re*_t_* (angle-averaged over all possible cross-flow directions). 

Equation (9) comes from CFD simulations based on the primitive continuity, momentum and scalar transport equations. Simulations were conducted at unit-cell (single fiber) scale, for regular hexagonal arrays of straight hollow fibers at ~50% of porosity with periodic boundary conditions imposed to all variables between opposite boundaries. Detailed results are reported in [25]. In the general case of mixed flow with both longitudinal (Re*_z_*) and transversal (Re*_t_*) Reynolds numbers being non-zero, mass transfer characteristics can be summarized by stating that Sh*_D_* was simply the larger between those computed for purely cross-flow at Re*_t_* and purely axial flow at Re*_z_*. This is reflected in Equation (9) which, for Re*_t_* = 0, provides the correct value computed for purely axial flow (Sh*_D_* = 9.85) at the current porosity.

The term *k_M_* in Equation (7) represents the membrane’s diffusive permeability for the solute considered which, in this work, is assumed to be urea. *k_M_* and other data relative to polyphenylene membranes, provided by the manufacturer, are listed in Table 1. 

On the shell side (dialysate), the porosity *ε* and the Darcy permeabilities *K_z_* and *K_t_* are experimentally measured and were equal to 51%, and 3.4 × 10^−10^ m^2^ and 3.2× 10^−11^ m^2^, respectively. On the lumen side (blood), the porosity was determined to be 29% and the measured axial permeability *K_z_* was 3.4 × 10^−10^ m^2^. These data are in fair agreement with those used by Ding et al. [22] for similar membranes. Since the lumen-side flow is laterally confined, *K_t_* is set to zero.

### 2.2. Geometries Investigated

In this work, different module geometries were simulated via computational fluid dynamics (CFD). Several cylindrical configurations sharing the same membrane area (1.7 m^2^), porosity (51%) and total volume (307 cm^3^), but differing in aspect ratio and inlet/outlet arrangement, were compared. The study was also extended to a couple of rectangular-shaped modules.

**Figure 2:**
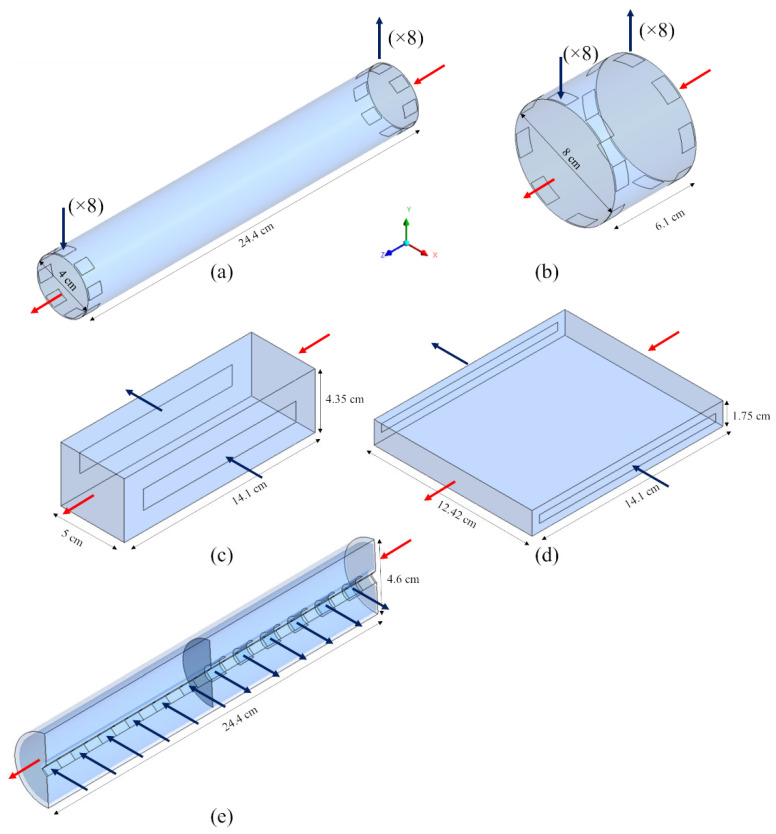
Three-dimensional computational domains for the simulated geometries: (**a**) long cylindrical; (**b**) short cylindrical; (**c**) thick rectangular; (**d**) flat rectangular; (**e**) coaxial cylindrical. Arrows: blood (red), dialysate (blue).

Figure 2 shows the computational domains of the modules simulated and the related dimensions. Arrows indicate the flow direction for blood (red) and dialysate (blue).

The first geometry simulated, Figure 2a, is representative of a typical commercial haemodialysis unit. This computational domain includes 8 inlets and 8 outlets for the dialysate flow, which simulate the presence of a fluid distributor, usually provided in the commercial modules. Figure 2b represents a modified cylindrical geometry, sharing with the previous one the volume of dialysate but differing in size. This computational domain, also provided by the dialysate inlet and outlet ports, is twice in diameter and a quarter in length compared to the previous one. In order to assess the potential benefit of high cross-flow velocities, with the possible enhancement of mass transport, two hypothetical rectangular geometries (Figure 2c,d) were also studied. These rectangular-shaped modules are purely in cross-flow, thanks to their slit inlet and outlet ports.

Finally, the geometry in Figure 2e represents a coaxial cylindrical hollow-fiber module of the type commercialized as Liqui-Cel^®^ for liquid–liquid or gas–liquid extraction [26]. For symmetry reasons, only one half of the computational domain was simulated, in order to reduce the computational load.

For some of the geometries in Figure 2a,b,e, alternative inlet–outlet arrangements were also simulated, for a total of 13 different configurations.

### 2.3. Computational Grids

Before the final simulations, a grid-independence analysis was conducted. The sensitivity of the results to the discretization degree was carefully tested: both hydrodynamics and mass transfer quantities (e.g., the pressure drops of the two fluids and the solute clearance) were compared as functions of the number of computational volumes. The inlet blood and dialysate flow rates were 300 mL/min and 500 mL/min, respectively, on the basis of the typical operating conditions. The ultrafiltration flow rate was set at 10 mL/min, as in the final simulations, whose results are presented in this work.

For the long cylindrical geometry, four grids were compared by increasing the total number of finite volumes (FV) from ~28,000 to ~890,000. A grid of ~280,000 finite volumes exhibited a maximum discrepancy with the finest grid lower than 1%, thus guaranteeing the practical independence of the results from the discretization degree. Therefore, grids with a similar discretization degree were adopted also for the other geometries. For each geometry, the same computational grid was used for both fluids.

Table 2 reports the main features of the grids used in the final simulations. For any geometry, the same grid was used independently of the inlet–outlet configurations. For all the geometries investigated, the computational grids were composed of hexahedral volumes only, known to provide more accurate results than tetrahedral or hybrid grids [25].

### 2.4. Inlet–Outlet Configurations

**Figure 3:**
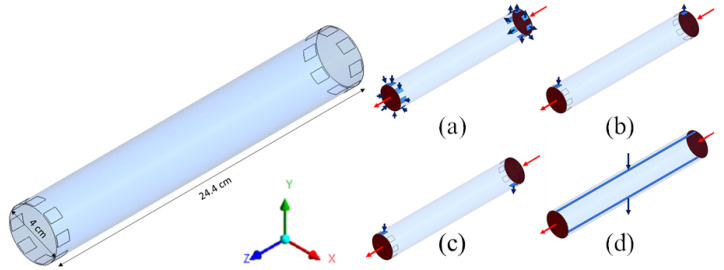
Three-dimensional computational domains for the long cylindrical geometry. Insets show the four configurations simulated: (**a**) 8 inlets and 8 outlets; (**b**) 1 inlet and 1 outlet on the same side; (**c**) 1 inlet and 1 outlet on opposite side; (**d**) 1 inlet and 1 outlet through longitudinal slits in the shell wall. Arrows: blood (red), dialysate (blue).

**Figure 4:**
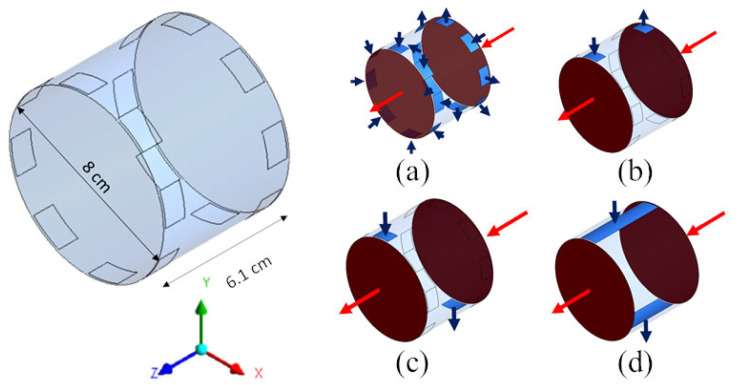
Three-dimensional computational domains for the short cylindrical geometry. Insets show the four configurations simulated: (**a**) 8 inlets and 8 outlets; (**b**) 1 inlet and 1 outlet on the same side; (**c**) 1 inlet and 1 outlet on opposite side; (**d**) 1 inlet and 1 outlet through longitudinal slits in the shell wall. Arrows: blood (red), dialysate (blue).

For both cylindrical geometries (a) and (b) of Figure 2, four configurations of the inlet and outlet ports of the dialysate were considered, as shown in Figure 3 for the “long cylindrical” geometry and in Figure 4 for the “short cylindrical” one: 8 inlets and 8 outlets (a), 1 inlet and 1 outlet on the same side (b), 1 inlet and 1 outlet on opposite sides (c), and 1 inlet and 1 outlet in the form of slits running the entire length of the module (d).

For the two rectangular geometries in Figure 2c,d, only one inlet–outlet configuration was considered.

**Figure 5:**
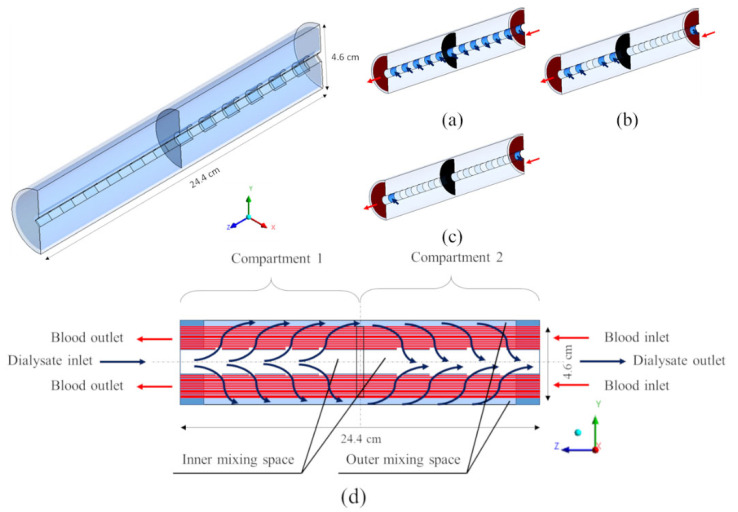
Three-dimensional computational domains for the coaxial cylindrical geometry. Insets show the three configurations simulated: (**a**) 6 inlets and 6 outlets; (**b**) 4 inlets and 1 outlet; (**c**) 1 inlet and 1 outlet. (**d**) Two-dimensional longitudinal cross-section view. Arrows: blood (red), dialysate (blue).

In the coaxial cylindrical geometry of Figure 2e, the path of the dialysate is more complex than the previous ones. This particular geometry, designed to better promote cross-flow conditions [26], consists of two axial compartments partially divided by a cross-sectional partition and connected to each other by an annular peripheral passage. As shown in Figure 5, the dialysate goes from the inner mixing space into the first compartment, where the hollow fibers are located and mass transfer takes place. Then, the dialysate passes into the second compartment through the annular peripheral passage (outer mixing space), and crosses the second half of the bundle before reaching the inner mixing space of the second compartment and leaving the dialysis unit. Three configurations were simulated, differing in the number and type of inlets and outlets (6 inlets and 6 outlets, 4 inlets and 1 outlet, and 1 inlet and 1 outlet; see graphs (a)–(c) in Figure 5).

### 2.5. Simulation Strategy

The simulation method requires an iterative procedure. In the model, blood and dialysate were identified as two different fluids flowing through two different porous media, but occupying the same module volume and exchanging both solution and solute fluxes between each other. 

Urea, a blood toxin, was chosen in this study as the main marker for the control of the dialysis treatment. For this solute, a molecular weight (MW) of 60 Da and a Staverman reflection coefficient (*σ*) equal to zero were used [27]. The physical properties of the fluids [28], together with the operating flow rates and inlet concentrations of urea used in the simulations, are reported in Table 3. For simplicity, the density of both blood and dialysate were set to 1000 kg m^−3^.

Imposed flow rates at the inlets and imposed pressures at the outlets were set as boundary conditions for both the blood and the dialysate. Specifically, the inlet blood flow rate was 300 mL/min and the dialysate one was 500 mL/min. Regarding pressures, the dialysate outlet pressure was set to zero (relative), while the blood outlet pressure was tuned so as to obtain a desired value of the ultrafiltration flow rate (10 mL/min). No slip walls with zero solute mass flux were set at the external surfaces of the module.

**Figure 6:**
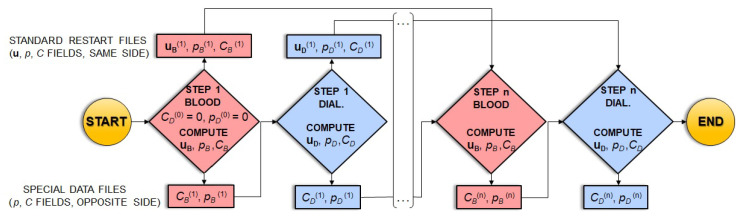
Flow chart of the computational procedure employed in the simulations.

The procedure, schematically illustrated by the flow chart in Figure 6, starts with the first iteration on the blood side (STEP 1 BLOOD). In this step, in the mass transfer source term *S_c_* of Equations (5) and (6) (written for blood, i.e., with the minus sign), the dialysate-side concentration *C_D_* and pressure *p_D_* are kept equal to zero. At the end of the simulation, the computed blood-side flow field **u***_B_*, pressure *p_B_* and concentration *C_B_* are written to the standard blood-side restart file of the code (upper row of the flow chart); *p_B_* and *C_B_* are also stored in a special data file (lower row of the flow chart).

In the subsequent simulation (STEP 1 DIAL.), in the mass transfer source term *S_c_* of Equations (5) and (6) (written for the dialysate, i.e., with the plus sign), the blood-side concentration *C_B_* and pressure *p_B_* are read in from the above-mentioned special data file. At the end of the simulation, the computed dialysate-side flow field **u**_D_, pressure *p_D_* and concentration *C_D_* are written to the standard dialysate-side restart file (upper row of the flow chart); *p_D_* and *C_D_* are also stored in a special data file (lower row of the flow chart). 

All the subsequent iterations restart from the standard restart file that contains the results of the previous iteration for the relevant side and read in the last available concentration and pressure distributions for the opposite side from the relevant special data file. 

Convergence is considered to have been reached when, for both the blood and the dialysate, the differences in outlet flow rates and solute concentrations between two consecutive steps become lower than 0.5%, i.e., when the clearance value reaches a *plateau*.

The overall parameter that usually allows the dialyzer efficiency to be quantified is the aforementioned solute clearance (CL), defined as:

(10) $$\mathrm{CL}=\frac{Q_{Bi}\;C_{Bi}-Q_{Bo}\;C_{Bo}\;}{C_{Bi}}$$

in which *Q_Bi_* and *Q_Bo_* are the blood flow rates at the inlet and outlet of the module, while *C_Bi_* and *C_Bo_* are the blood inlet and outlet solute concentrations.

**Figure 7:**
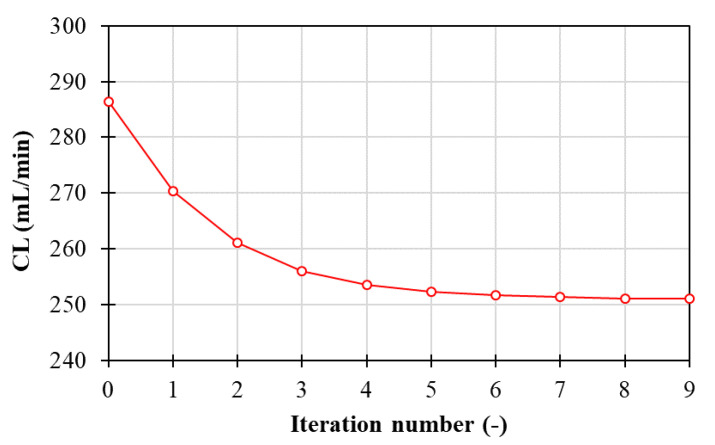
Clearance as a function of the number of iterations.

This parameter, being itself dependent on blood-side flow rates and solute concentrations, can be used to evaluate the convergence of the iterative procedure. Figure 7 reports the solute clearance as a function of the number of iterations for the long cylindrical geometry with 1 inlet and 1 outlet on the same side.

The solute clearance decreases as the number of iterations increases, and reaches, in this case, an almost constant value between the 9th and 10th steps of the simulation. Qualitatively analogous behaviors are also obtained for all the simulations regarding the other geometries investigated in the present work; for the sake of brevity, the plots are not reported. In all cases, convergence was achieved in 6–10 steps per side. 

## 3. Results and Discussion

### 3.1. Model Validation

Experimental values of clearance were used for model validation. They were determined at the Medtronic^®^ laboratories for the PHYLTHER^®^ HF 17SD module (membrane area 1.7 m^2^ and membrane properties as summarized in Table 1) according to the ISO 8637-1:2017 guidelines, which prescribe the use of a saline solution instead of blood. Therefore, the simulations purposely conducted for the model validation were carried out with a fluid that has the physical properties of the dialysate (Table 3) in both compartments and by setting the relevant oncotic pressure to zero. 

In order to test the robustness of the model to higher molecular weight solutes, B12 vitamin (MW = 1355 Da) was also considered for validation purposes. For B12 vitamin, the membrane’s diffusive permeability *k_M_* = (3.1 ± 0.2) × 10^−6^ m/s was provided by the manufacturer, and the solute diffusivities in blood [29] and in dialysate [30] were set as in the relevant literature.

Table 4 compares the experimental results and model predictions for the clearance of urea and B12 vitamin at different lumen-side flow rates (200, 300 and 400 mL/min), at a dialysate flow rate of 500 mL/min and an ultrafiltration flow rate of 10 mL/min. Experimental data were obtained as averages over 29 test cases and are reported together with the respective standard deviation (STD). 

As expected, the clearance increases as the lumen-side flow rate increases. In all cases, model predictions fall within the dispersion interval of the corresponding experimental data. For both solutes, the maximum discrepancy with the experimental data is less than 3%, thus indicating a good or even excellent agreement.

### 3.2. Comparison of Model Predictions

Simulations were conducted for all the configurations described in Section 2.2, each characterized by the geometry of the computational domain and by the number and location of the inlet and outlet openings. 

According to the classic theory of mass or heat exchangers, for any overall transfer coefficient *U* and transfer area, the configuration yielding the highest transfer efficiency and, thus, the highest clearance CL is the ideal counter-flow one. In fact, the standard configuration of commercial hemodialyzers (Case 1A of Table 5) closely approaches the counter-flow design, save for small cross-flow regions near the inlets and outlets. However, in regular fiber arrays, the shell-side Sherwood number Sh*_D_* is a sensitive function of the transverse Reynolds number (see Equation (9)) and, thus, may significantly increase in cross-flow, also making *U* increase. Therefore, in principle, it would be possible to depart from the counter-flow design, while still keeping the clearance values high, thanks to the increase in Sh*_D_* and *U* due to the cross-flow. One of the main motivations of the present study was the wish to investigate this possibility.

For each configuration, Table 5 reports the main performance parameters: pressure drops Δ*p_B_* and Δ*p_D_* in blood and dialysate, shell-side average Sherwood number 〈Sh*_D_*〉, overall urea clearance CL and percentage contribution of ultrafiltration to mass transport UF.

The lumen-side and shell-side pressure drops Δ*p_B_* and Δ*p_D_* were calculated as:

(11) $$\Delta p_{B}=p_{B,i}-p_{B,o}$$

(12) $$\Delta p_{D}=p_{D,i}-p_{D,o}$$

in which the pressures at the inlet of the module, *p_B,i_* and *p_D,i_*, were the area-weighted averages of pressure on the inlet surface and *p_B,o_* and *p_D,o_* were the outlet pressures imposed for blood and dialysate, respectively, as mentioned in Section 2.5. Therefore, Δ*p_B_* and Δ*p_D_* include only the pressure loss in the computational domains and do not account for the pressure losses in the end manifolds of the module, which can be a significant fraction of the overall losses, as recently reported by Karabelas et al. [31,32]. In fact, manifolds are not included in the present computational domains.

With regard to the shell-side pressure drop, largely different results are obtained depending on the geometry and the inlet–outlet arrangement. 

In general, the lowest pressure drops are provided by the configurations in which the dialysate flow travels the shortest distance from inlet to outlet. This occurs in most cases when the main flow is orthogonal to the fiber bundle; the lowest value (~918 Pa) is obtained for the short cylindrical geometry with eight inlet–outlet openings (Case 2A), in which the module length is very small. The second lowest value (~1700 Pa) is obtained for the coaxial cylindrical geometry with six inlets and six outlets (Case 4A).

The highest pressure drops are obtained in those configurations in which the dialysate path is longest and the inlet–outlet area is lowest, namely in the long cylindrical geometry with one inlet and one outlet (Cases 1B and 1C), which yields Δ*p_D_* ≈ 16300 Pa almost independently of the relative position of these openings. The large difference between the pressure drop values obtained for the same geometry but with one or eight inlets and outlets shows that only about one half of the pressure drop occurs in the fiber bundle, while the remaining part is localized in the expansion and contraction regions close to the openings (explicitly included in the computational domain). These configurations are followed by the short geometry with one inlet and one outlet on opposite sides, Case 2C, yielding Δ*p_D_* ≈ 10950 Pa. A large Δ*p_D_* also characterizes the flat rectangular geometry (Case 3B).

With regard to the shell-side Sherwood number, the values obtained are less scattered, ranging from ~13 to ~25. The lowest value (13.4) is obtained for the short cylindrical configuration with one inlet and one outlet, Case 2B, in which most of the flow is parallel to the fiber bundle. The highest Sh*_D_* (24.9) is obtained for the flat rectangular geometry with the slit inlet–outlet, Case 3B, in which the dialysate travels in a cross-flow with respect to the fiber bundle and, due to the small passage area, the velocity is relatively high.

Clearance values are, in most cases, little affected by the configuration of the module, and range between ~227 and ~257 mL/min. The only two exceptions are the short cylindrical geometry with one inlet and one outlet on the same side, Case 2B, in which the flow follows a short path between the inlet and outlet, bypassing most of the fiber bundle volume, and whereby a very low clearance (~72 mL/min) is obtained. The other exception is Case 2A, which exhibits a clearance of ~178 mL/min: as the concentration maps will show (Section 3.3), the middle region of the module is not involved in the mass transfer process, thus indicating a poor utilization of the membrane surface area.

The last column in Table 5 reports the percent contribution UF of ultrafiltration to mass transport and, thus, to clearance. For a given ultrafiltration solution flux (a quantity set to 10 mL/min in all the present configurations by adjusting the outlet pressure at the blood side), the ultrafiltration component of the solute mass flux depends on the details of the combined distributions of (i) the transmembrane pressure and (ii) the solute concentrations in the two fluids, but not on the transmembrane concentration *difference*, while the diffusive solute transport is proportional to this difference. Therefore, the configurations characterized by largely different diffusive solute transport rates may exhibit comparable ultrafiltration solute transport rates, so that the relative contribution UF tends to be highest when CL is lowest.

In summary, simulation results show that, with few exceptions, the module configurations sharing the same membrane area and the same flow rates, but differing in aspect ratio and inlet/outlet arrangement, yield very different pressure drops, moderately different average shell-side Sherwood numbers and values of the clearance that never exceed the values obtained with the commercial module, with small percentage contributions (~2–5%) of ultrafiltration to this last quantity.

**Figure 8:**
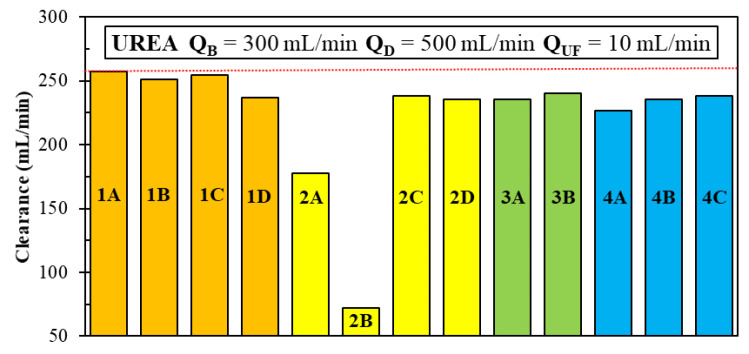
The predicted values of urea clearance for all the module configurations investigated. Color of the bars: orange (long cylindrical); yellow (short cylindrical); green (rectangular); azure (coaxial cylindrical).

Figure 8 summarizes in graphical form the predicted urea clearances for the various module configurations simulated.

### 3.3. Dialysate Velocity and Solute Concentration Distributions

**Figure 9:**
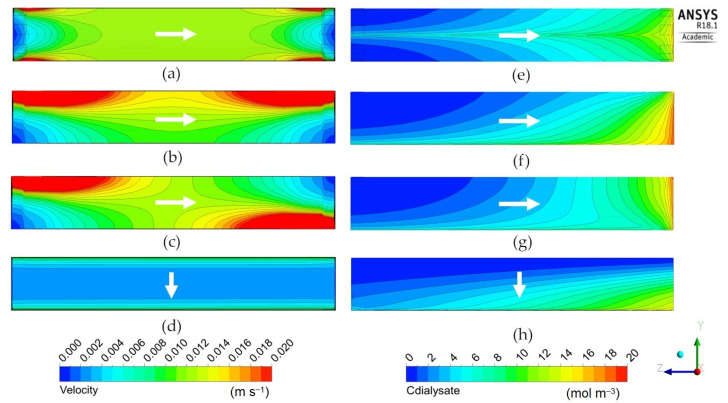
Dialysate velocity module (**left**) and urea concentration (**right**) distributions in the *zy* mid-plane for the long cylindrical geometry. Inlet and outlet openings configuration: (**a**,**e**) 8 inlets and 8 outlets; (**b**,**f**) 1 inlet and 1 outlet on the same side; (**c**,**g**) 1 inlet and 1 outlet on opposite sides; (**d**,**h**) 1 inlet and 1 outlet through longitudinal slits in the shell wall. The white arrow indicates the main dialysate flow direction; blood flows from right to left in all cases.

**Figure 10:**
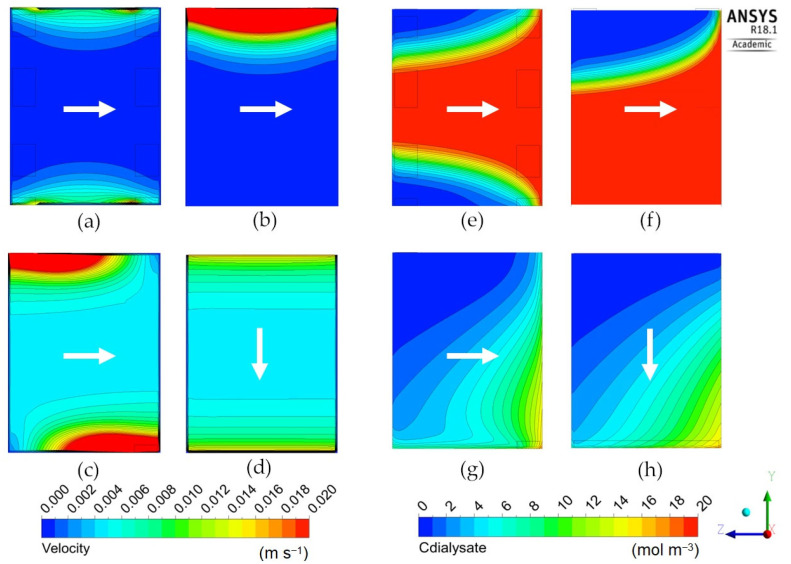
Dialysate velocity module (**left**) and urea concentration (**right**) distributions in the *zy* mid-plane for the short cylindrical geometry. Inlet and outlet openings configuration: (**a**,**e**) 8 inlets and 8 outlets; (**b**,**f**) 1 inlet and 1 outlet on the same side; (**c**,**g**) 1 inlet and 1 outlet on opposite sides; (**d**,**h**) 1 inlet and 1 outlet through longitudinal slits in the shell wall. The white arrow indicates the main dialysate flow direction; blood flows from right to left in all cases.

**Figure 11:**
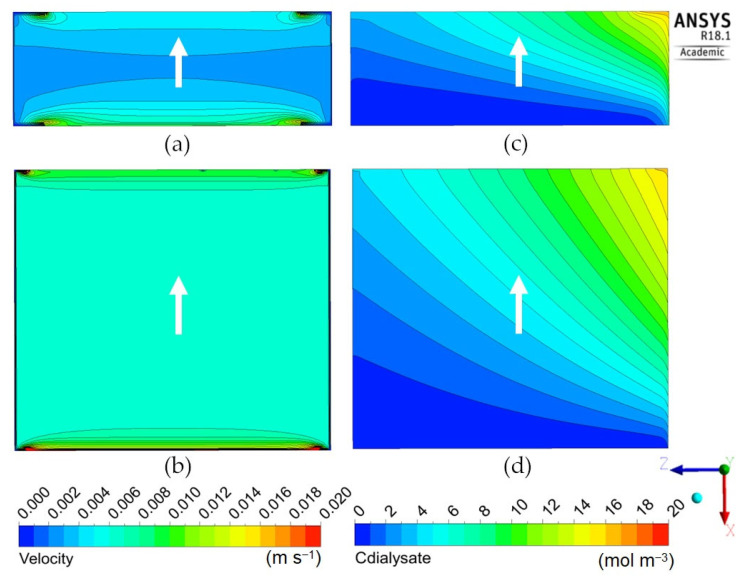
Dialysate velocity module (**left**) and urea concentration (**right**) distributions in the *zx* mid-plane for the rectangular geometries: (**a**,**c**) thick; (**b**,**d**) flat. The white arrow indicates the main dialysate flow direction; blood flows from right to left in all cases.

The following Figure 9, Figure 10 and Figure 11 report the predicted dialysate-side distributions of the velocity module (left) and urea concentrations (right) in a module’s mid-plane for the long cylindrical, short cylindrical and rectangular configurations. In all plots, the blood flow is from right to left, whereas the main dialysate flow direction is indicated by an arrow (note that most configurations are in counter-flow but some, namely Cases 1D, 2D, 3A and 3B in Table 5 and Figure 8, are in cross-flow).

Figure 9 is for the “long cylindrical” geometry 1 in its four variants, 1A–1D. It shows that the standard configuration 1A with eight inlets and eight outlets, maps (a) and (e), exhibits a rather uniform distribution of the fluid’s velocity module, but also a marked radial gradient of the solute concentration, superimposed on the main axial gradient. 

Very similar maps of velocity and concentration were obtained by Ding et al. [22,23], who stated that these profiles are induced by the dialysate inlet/outlet ports. Concentration distributions qualitatively similar to that reported in map (e), exhibiting higher values in the module centerline than in the periphery, were also reported by Liao et al. [21]. By using X-ray computed tomography, Frank et al. [33] also obtained maps exhibiting lower concentrations in the outer portion of the module and a region at higher concentration close to the center line. Maps comparable to those reported in the present work were also obtained experimentally by Osuga et al. [17], who used contrast-enhanced magnetic resonance imaging (MRI) to investigate the dialysate flow in hollow-fiber dialyzers.

The two configurations 1B and 1C, maps (b)–(f) and (c)–(g), characterized by a single inlet and a single outlet (placed either on the same side or on opposite sides of the module) exhibit a strongly non-uniform distribution of velocity and a significant side-to-side solute concentration gradient. Configuration 1D, maps (d) and (h), characterized by a slit inlet and outlet, exhibits a very uniform velocity distribution, while in the solute concentration a strong lateral gradient dominates the axial one, due to the axial blood flow. The clearances are similar for Cases 1A, 1B and 1C, while Case 1D exhibits a slightly lower clearance (237 mL/min against 251–257 mL/min) and also a smaller contribution of ultrafiltration (3.64% against 4.10–4.70%), indicating a minor effectiveness of the cross-flow arrangement. Due to the lack of experimental data or modelling results for non-conventional configurations of the module, the model predictions presented in this work cannot be compared with the literature.

Figure 10 is for the “short cylindrical” geometry 2 in its four variants 2A–2D (see Table 5). Configuration 2A, with eight inlets and eight outlets, maps (a) and (e), exhibits a highly non-uniform distribution of the fluid’s velocity module: the core region near the axis is practically motionless, while the fluid flows mainly in the outer region of the cylindrical module. As a consequence, the whole central region of the dialysate volume attains the maximum possible solute concentration (practically identical to the inlet blood concentration, i.e., 20 mol/m^3^) and thus does not take any part in the diffusive mass transfer process, causing a low overall clearance value (178 mL/min). 

The situation is even worse in configuration 2B with one inlet and one outlet on the same side, maps (b) and (f), in which a shortcut from the inlet to the outlet occurs for the dialysate flow. This causes a fraction of the dialysate even larger than in Case 2A to be still, thus attaining the maximum possible solute concentration and taking no part in mass transfer. The consequence is the least clearance value of all cases (72 mL/min). Note that the same case is also characterized by the highest relative contribution of ultrafiltration to clearance (12.8%), simply because the associated mass flux, unlike its diffusive counterpart, is not affected by the concentration difference between the blood and dialysate.

To the contrary, configuration 2C, maps (c) and (g), also characterized by a single inlet and a single outlet, but placed on opposite sides, exhibits a relatively uniform velocity distribution (with the exception of the immediate neighborhood of the inlet–outlet openings), dominated by the cross-flow component. This causes a regular distribution of the solute concentration, which exhibits axial and lateral gradients of comparable values, and results in a relatively high value of the clearance (238 mL/min), much larger than in Cases 2A and 2B, and comparable with those of the “long cylindrical” configurations.

Finally, configuration 2D, maps (d) and (h), bearing slit inlet and outlet openings and thus a purely cross-flow velocity field, presents a rather uniform velocity distribution, a distribution of the solute concentration that is regular and very similar to that of the previous Case 2C in maps (c)–(g), and about the same value of the clearance (236 mL/min).

**Figure 12:**
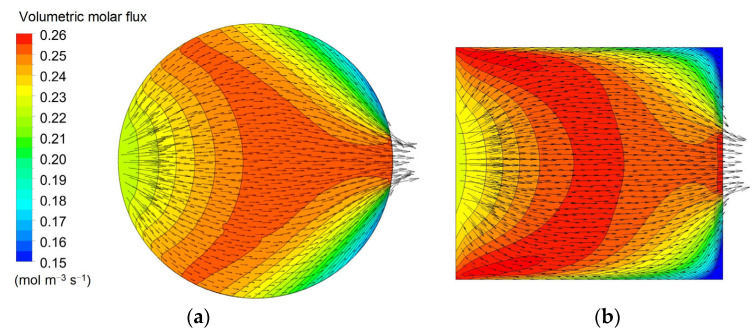
Comparison of vector plots of the in-plane dialysate velocity and distributions of the solute’s volumetric molar flux in a cross-section orthogonal to the fiber bundle for two configurations: (**a**) 2D (“short cylindrical” with slit inlet–outlet); (**b**) 3A (“thick rectangular”).

Figure 11 is for the “rectangular” geometry 3 in its two variants 3A (thick) and 3B (flat), see Table 5. Both configurations, especially the “flat” one, maps (b) and (d), exhibit a rather uniform velocity distribution (the local peaks visible near the corners of the inlet and outlet openings are a consequence of the imposed hydraulic anisotropy of the porous medium). The solute concentration exhibits the expected, regular, diagonal stratification typical of a cross-flow. The clearance is similar to that predicted for the configurations 1D and 2D which, too, present slit inlet–outlet openings and a cross-flow velocity distribution. The reason for this similarity is that, due to the porous medium modelling of the dialysate compartment, the fluid “forgets” the inlet flow non-uniformity and spreads evenly through all the available volume independently of the exact inlet velocity profile and geometry of the module. For example, Figure 12 shows the in-plane velocity vectors and maps of the solute volumetric molar flux, i.e., the source term *S_C_* as defined by Equations (5) and (6), in a cross-section of Cases 2D (“short cylindrical” with slit inlet–outlet) and 3A (“thick rectangular”, also with slit openings). The similarity of both the velocity and solute flux distributions is evident.

**Figure 13:**
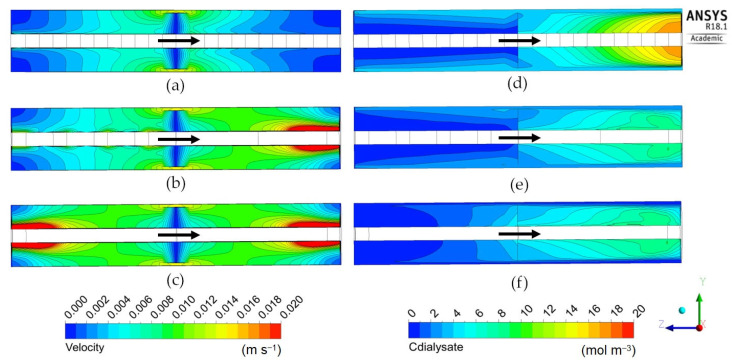
Dialysate velocity module (**left**) and urea concentration (**right**) distributions in the *zy* mid-plane for the coaxial cylindrical geometry. Inlet and outlet openings configuration: (**a**,**d**) 6 inlets and 6 outlets; (**b**,**e**) 4 inlet and 1 outlet; (**c**,**f**) 1 inlet and 1 outlet. The arrow indicates the main dialysate flow direction; blood flows from right to left in all cases.

Finally, Figure 13 regards the “coaxial cylindrical” geometry 4 with its three variants 4A–4C in Table 5, differing in the number and location of inlet and outlet openings. These configurations provide intermediate values of clearance (227–238 mL/min), with some differences between the three types. 

Type 4A, maps (a) and (d), with six inlets and six outlets, yields a rather uniform and symmetric velocity distribution and a high solute concentration in the dialysate at the module’s right end. However, the outlet concentration averaged over all six outlets is relatively low, yielding the lowest clearance value among the three variants (227 mL/min). 

Type 4B, maps (b) and (e), with four inlets and one outlet, exhibits the most non-uniform and most asymmetric velocity distribution, but also a rather uniform concentration, and provides a higher clearance (236 mL/min) than type 4A. The best performance in terms of clearance is provided by type 4C, maps (c) and (f), with one inlet and one outlet, which exhibits a concentration distribution very similar to that of the previous case, Case 4B. The velocity distribution is symmetric as in Case 4A, but exhibits strong peaking factors at both ends of the module. 

The overall dialysate-side pressure drop increases strongly from Case 4A to Case 4C; the contribution of ultrafiltration to clearance ranges between ~3% and ~4%, and is highest in Case 4A.

## 4. Conclusions

Several alternative geometries of hemodialysis modules were simulated by means of a computational fluid dynamics model. This is based on a porous media treatment and accounts for both diffusive and convective (ultrafiltration) fluxes. The hydrodynamic properties from measurements and the mass transfer characteristics from analytical solutions (lumen side) and CFD single-fiber (unit-cell) simulations (shell side) were used. Modules with a membrane area of 1.7 m^2^ and a bundle porosity of 0.51 were considered. The membrane’s hydraulic and diffusive permeabilities were representative of commercial polyphenylene membranes, as provided by the manufacturer. Model predictions, for the commercial geometry, were validated for two solutes, urea and B12 vitamin, and three blood flow rates (200, 300 and 400 mL/min), exhibiting a fair agreement with experimental data (maximum discrepancy < 3%).

The simulated configurations differed in geometry and flow path, including both mainly longitudinal and mainly transverse flow. At fixed blood and dialysate flow rates (300 and 500 mL/min, respectively), and ultrafiltration flow rate of 10 mL/min, they exhibited much different dialysate-side flow fields and concentration distributions, resulting in different values of the clearance.

Although the Sherwood number increased in almost all the alternative configurations investigated (from ~14 in Case 1A to ~25 in Case 3B), this did not result in an increase in the amount of solute clearance compared to the commercial hemodialyzer module (Case 1A). Therefore, the present work gave a negative answer to the question of whether alternative geometries may enhance the module performance in terms of clearance. The reason lies in the small contribution given by the shell-side convective resistance (proportional to Sh^−1^) to the overall mass transfer resistance. Despite some alternative configurations, e.g., the “rectangular” cross-flow Cases 3A and 3B, exhibited Sherwood numbers much higher than that of the standard Case 1A, they were characterized by lower values of clearance (235–240 mL/min for Cases 3A and 3B vs. 257 mL/min for Case 1A) due to the significant deviation from the counter-flow design.

Therefore, it can perhaps be stated that existing dialyzers already lie close to a performance maximum in terms of configurations and operating conditions, so that the effect of changes in these variables can only be detrimental. The room for performance improvements, at least in terms of clearance, is probably very limited until a novel generation of membranes exhibiting a significantly larger diffusive permeability will appear on the stage.

## Figures and Tables

**Table 1:** Membrane data used in the present simulations.

Membrane Data	
Internal diameter of the hollow fibers, *d_i_* (μm)	200 ± 20
Thickness of the hollow fibers, *s* (μm)	30 ± 5
Diffusive permeability (for urea), *k_M_* for urea (m s^−1^)	(1.1 ± 0.2) × 10^−5^
Hydraulic permeability, *L_p_* (m s^−1^ Pa^−1^)	(6.6 ± 0.4) × 10^−11^

**Table 2:** Main features of the computational grids for the geometries investigated.

Geometry	Total Number of FV	Number of FV in the Cross-Sectional Plane
Long cylindrical	280,800	10,800
Short cylindrical	302,400	8400
Thick rectangular	301,050	10,035 (*zy* plane)
Flat rectangular	354,816	2772 (*zy* plane)
Coaxial cylindrical	299,520	11,520

**Table 3:** Fluids and urea properties used in the present simulations (37 °C).

Fluid	Density (kg m^−3^)	Viscosity (Pa s)	Diffusivity of Urea (m^2^ s^−1^)	Inlet Flow Rate (mL min^−1^)	Inlet Concentration (mol m^−3^)
Blood	1000	3.50 × 10^−3^	7.4 × 10^−10^	300	20
Dialysate	1000	7.62 × 10^−4^	1.8 × 10^−9^	500	0

**Table 4:** Experimental values and model predictions of urea and B12 vitamin clearance (mL/min) for PHYLTER^®^ HF 17SD [24].

Solute	*Q_B,i_* (mL min^−1^)	Exp. ^1^ (mL min^−1^)	Model (mL min^−1^)	Error
Urea	200	191 ± 7.0	192	0.5%
	300	249 ± 10	255	2.4%
	400	294 ± 14	294	0%
B12 vitamin	200	147 ± 8.0	144	2.0%
	300	164 ± 12	168	2.4%
	400	186 ± 16	182	2.2%

^1^ mean CL ± STD.

**Table 5:** Summary of the model predictions for the various geometries investigated.

**Long Cylindrical**						
**Case**	**Notes**	**Δ*p_B_* (Pa)**	**Δ*p_D_* (Pa)**	**〈Sh*_D_*** **〉**	**CL (mL/min)**	**UF (%)**
1A	8 inlets/8 outlets	9833	4510	14.1	257	4.10
1B	1 inlet/1 outlet s.s.	9833	16,280	17.5	251	4.70
1C	1 inlet/1 outlet o.s.	9833	16,320	18.2	255	4.30
1D	Slit inlet/outlet	9833	1812	19.8	237	3.64
**Short Cylindrical**						
**Case**	**Notes**	**Δ*p_B_* (Pa)**	**Δ*p_D_* (Pa)**	**〈Sh*_D_*** **〉**	**CL (mL/min)**	**UF (%)**
2A	8 inlets/8 outlets	635.0	917.5	15.0	178	3.64
2B	1 inlet/1 outlet s.s.	635.0	7134	13.4	72	12.8
2C	1 inlet/1 outlet o.s.	635.0	10,942	22.5	238	2.21
2D	Slit inlet/outlet	635.0	6026	22.7	236	2.28
**Rectangular**						
**Case**	**Notes**	**Δ*p_B_* (Pa)**	**Δ*p_D_* (Pa)**	**〈Sh*_D_*** **〉**	**CL (mL/min)**	**UF (%)**
3A	Thick	3347	2204	20.6	235	2.55
3B	Flat	3347	10,352	24.9	240	3.43
**Coaxial Cylindrical**						
**Case**	**Notes**	**Δ*p_B_* (Pa)**	**Δ*p_D_* (Pa)**	**〈Sh*_D_*** **〉**	**CL (mL/min)**	**UF (%)**
4A	6 inlets/6 outlets	8132	1694	16.6	227	4.29
4B	4 inlets/1 outlet	8132	3857	16.3	236	3.42
4C	1 inlet/1 outlet	8132	5614	15.9	238	3.80
